# Supplementary material for: Cross-platform normalization enables machine learning model training on microarray and RNA-seq data simultaneously
Source: Commun Biol. 2023 Feb 25;6:222. doi: 10.1038/s42003-023-04588-6 (PMC9968332; doi:10.1038/s42003-023-04588-6)
Supplement: Supplementary file 2 — Supplementary Information [file 42003_2023_4588_MOESM2_ESM.pdf]

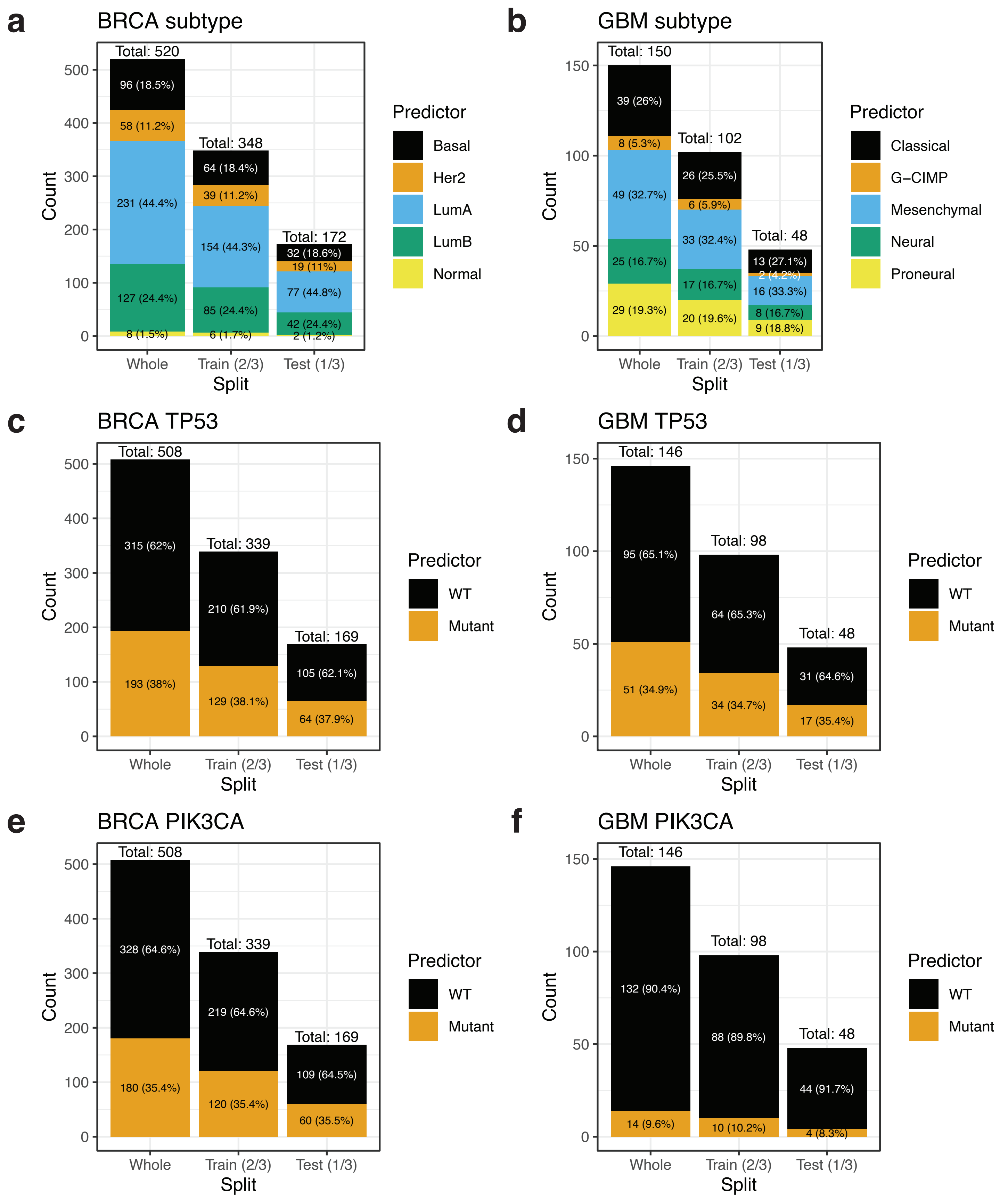

Supplementary Figure 1. Predictor class balance in the whole set (all samples) , training set (two-thirds), and test set (one-third). (a) BRCA subtype. (b) GBM subtype. (c) BRCA *TP53* mutation status. (d) GBM *TP53* mutation status. (e) BRCA *PIK3CA* mutation status. (f) GBM *PIK3CA* mutation status.

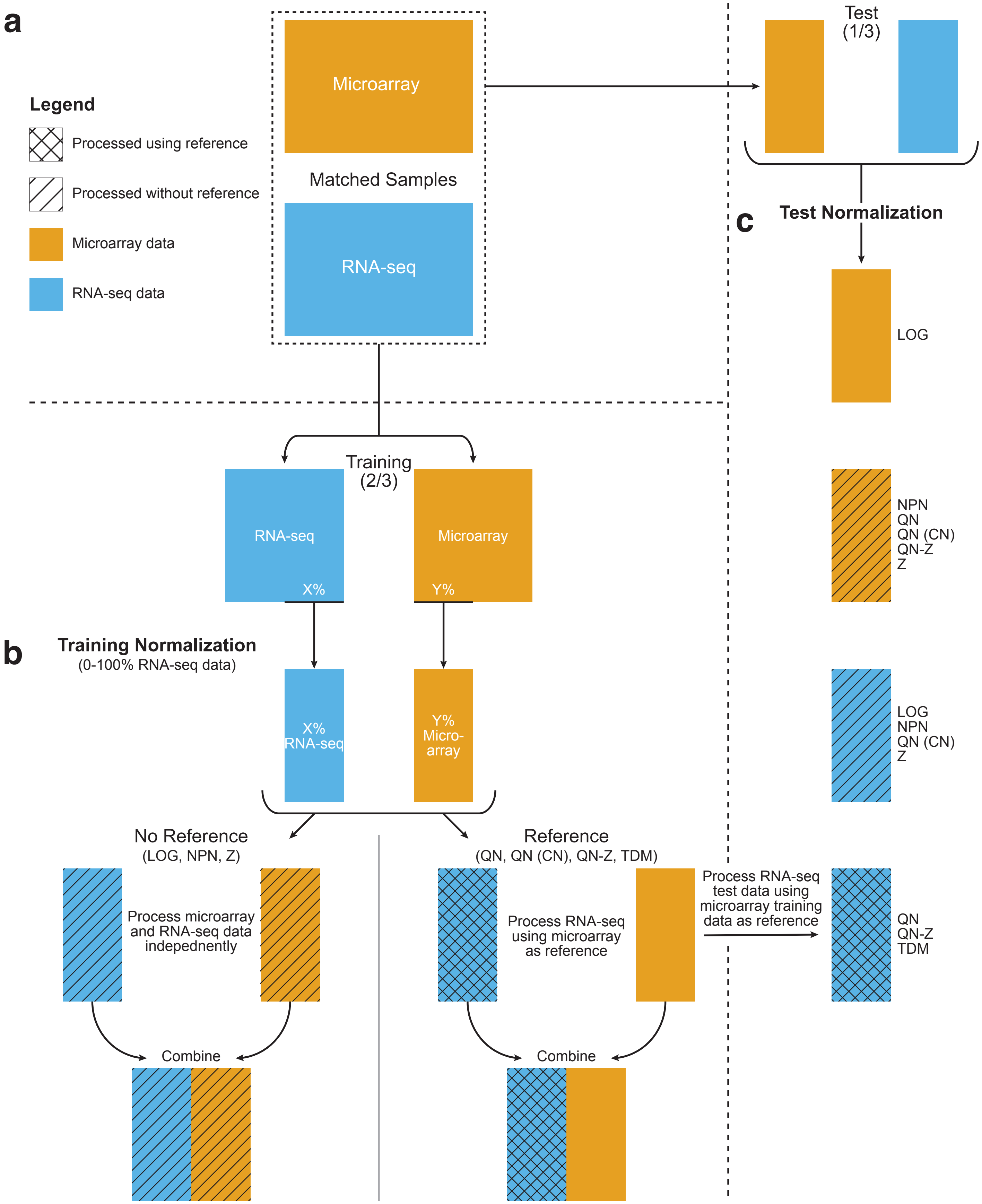

Supplementary Figure 2. Overview of cross-platform normalization with and without reference. (a) Matched samples are split into a training set and testing set. (b) Each training set is composed of between 0% and 100% RNA-seq data. For No Reference, the microarray portion and RNA-seq portion are normalized separately for LOG, NPN, and Z. With Reference, the microarray portion remains unprocessed (LOG) while the RNA-seq portion is normalized using the microarray portion as a reference distribution for QN, QN (CN), QN-Z, and TDM. Microarray and RNA-seq portions are then combined to form each cross-platform normalized training data set. (c) Microarray test data normalization occurs without reference for LOG, NPN, QN, QN (CN), QN-Z, and Z, and without reference for RNA-seq data for LOG, NPN, QN (CN), and Z. RNA-seq test data normalization uses microarray training data as the reference distribution for QN, QN-Z, and TDM at each percentage of RNA-seq titration in training. (LOG - log2-transformed; NPN – nonparanormal normalization; QN – quantile normalization; QN (CN) – quantile normalization with CrossNorm; QN-Z – quantile normalization followed by z-score; TDM – Training Distribution Matching; UN – untransformed; Z – z-score)

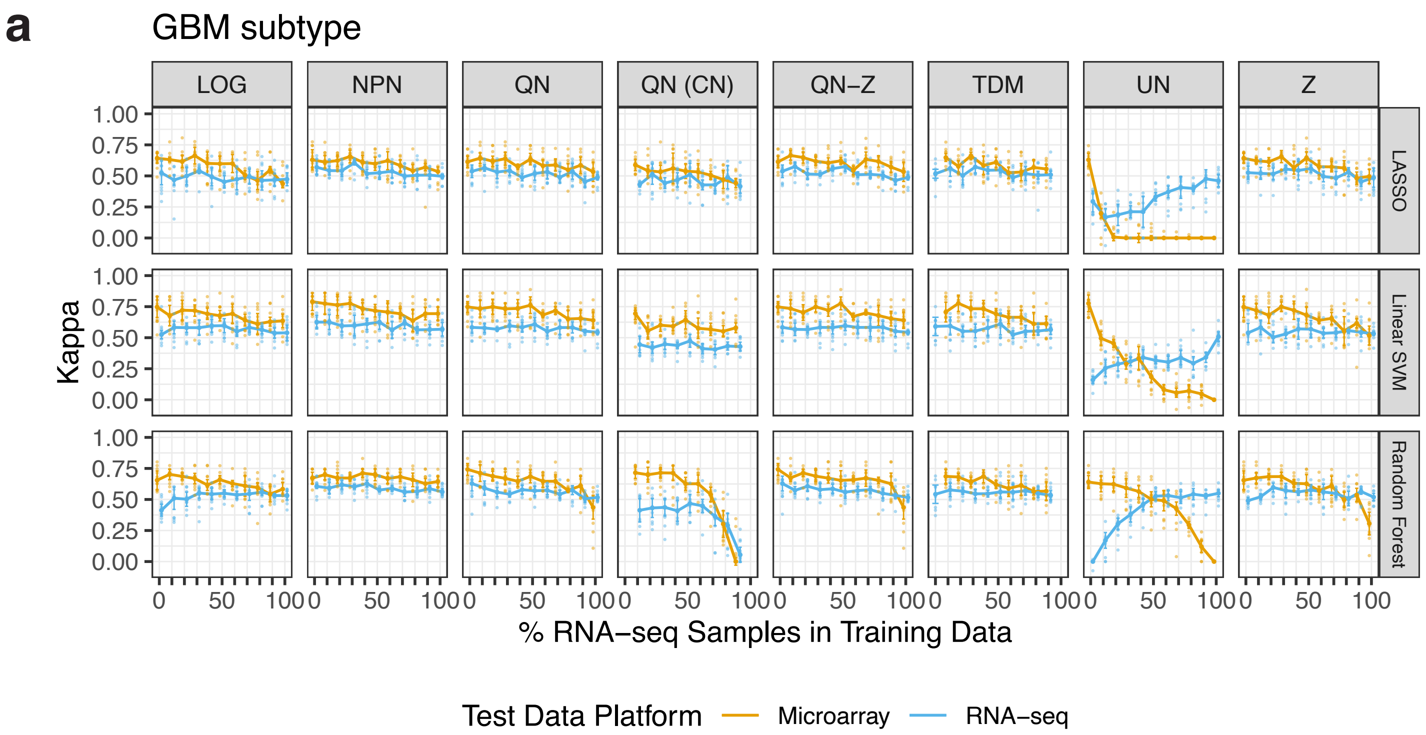

Supplementary Figure 3. GBM subtype classifier performance on microarray and RNA-seq test data. (a) Median Kappa statistics from 10 repeats of steps 1-3A from Figure 1 and for seven normalization methods (and untransformed data) are displayed. Median values are shown as points, and approximate 95% confidence intervals are shown around each median defined as +/- 1.58*IQR/sqrt(n) with IQR = interquartile range and n = number of observations.[60] (LOG - log2-transformed; NPN – nonparanormal normalization; QN – quantile normalization; QN (CN) – quantile normalization with CrossNorm; QN-Z – quantile normalization followed by z-score; TDM – Training Distribution Matching; UN – untransformed; Z – z-score)

a BRCA TP53

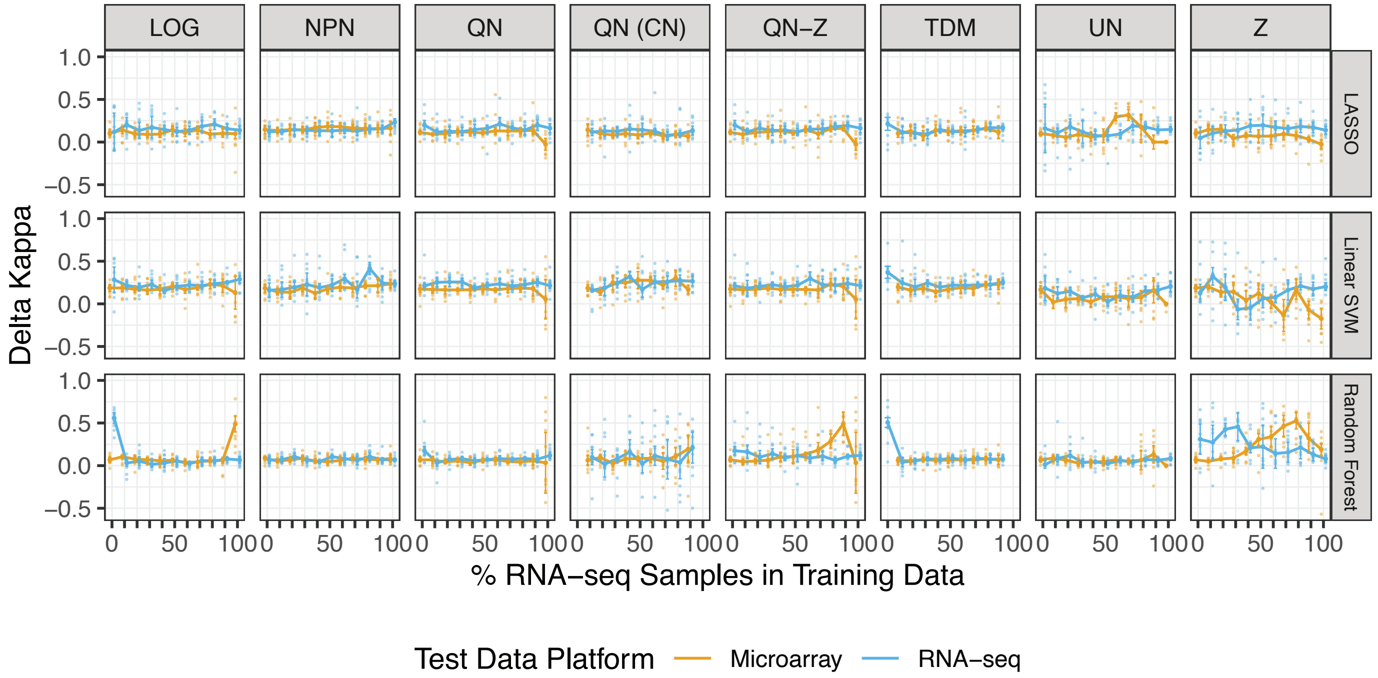

Supplementary Figure 4. BRCA *TP53* mutation classifier performance on microarray and RNA-seq test data. (a) Median delta Kappa statistics from 10 repeats of steps 1-3A from Figure 1 and for seven normalization methods (and untransformed data) are displayed. Median values are shown as points, and approximate 95% confidence intervals are shown around each median defined as +/- 1.58*IQR/sqrt(n) with IQR = interquartile range and n = number of observations.[60] Delta Kappa measures the difference in Kappa values between a null model and a model built with true labels. (LOG - log2-transformed; NPN – nonparanormal normalization; QN – quantile normalization; QN (CN) – quantile normalization with CrossNorm; QN-Z – quantile normalization followed by z-score; TDM – Training Distribution Matching; UN – untransformed; Z – z-score)

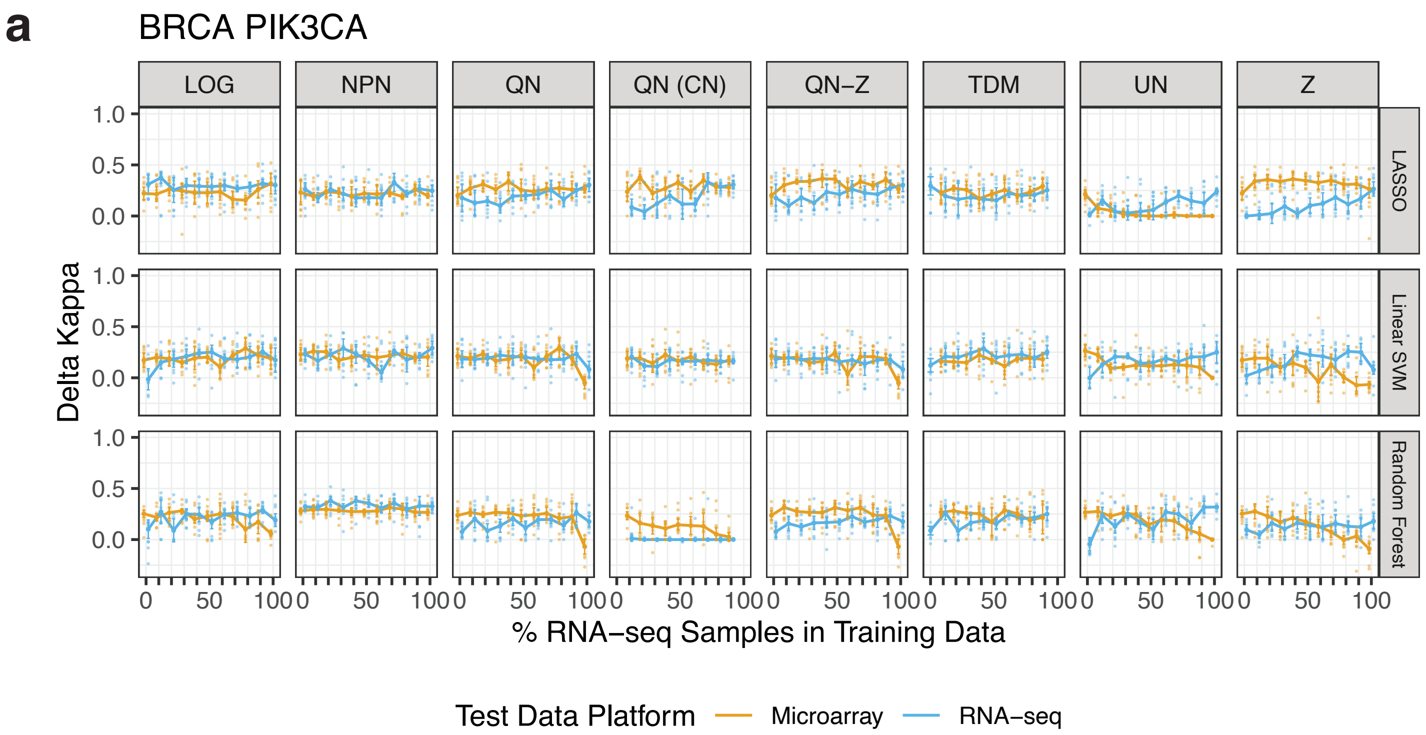

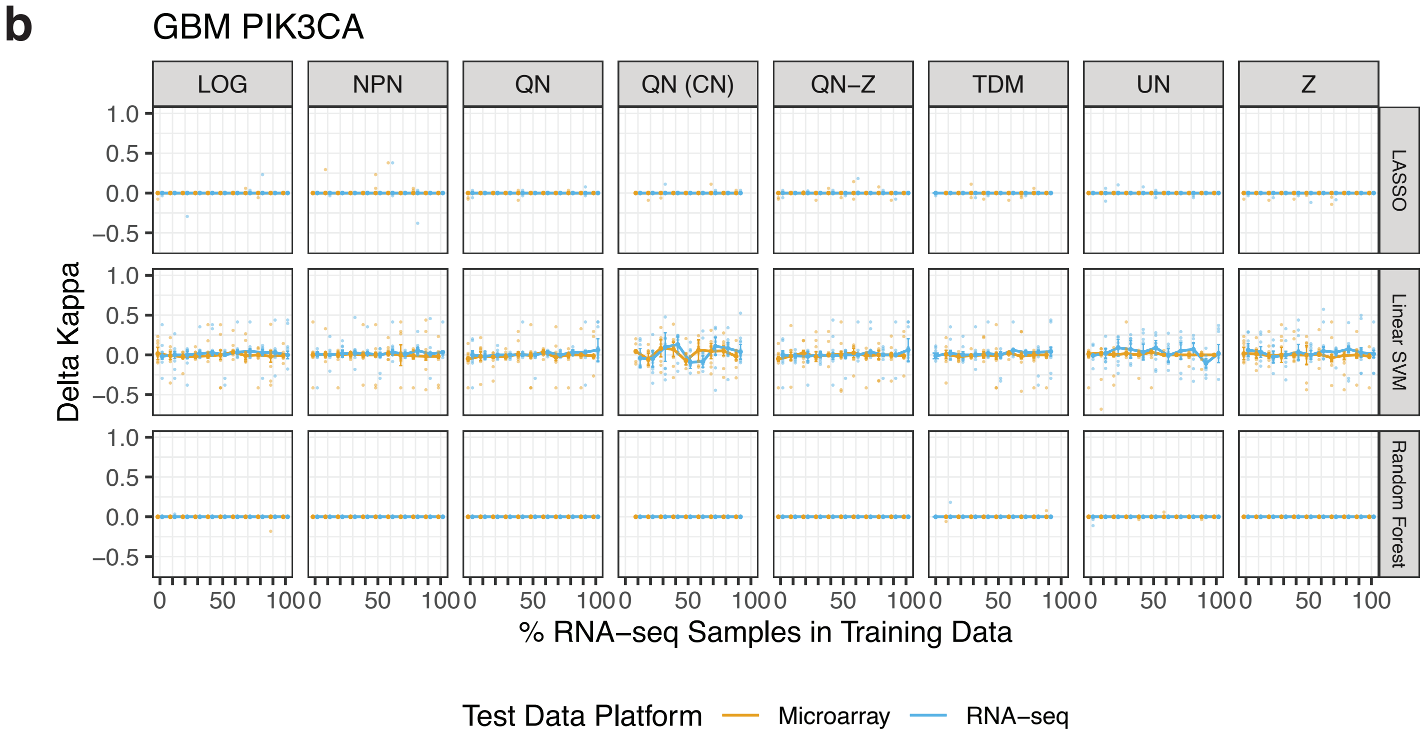

Supplementary Figure 5. Mutation classifier performance on microarray and RNA-seq test data in (a) BRCA *PIK3CA* and (b) GBM *PIK3CA*. Median delta Kappa statistics from 10 repeats of steps 1-3A from Figure 1 and for seven normalization methods (and untransformed data) are displayed. Median values are shown as points, and approximate 95% confidence intervals are shown around each median defined as +/- 1.58*IQR/sqrt(n) with IQR = interquartile range and n = number of observations.[60] Delta Kappa measures the difference in Kappa values between a null model and a model built with true labels.(LOG - log2-transformed; NPN – nonparanormal normalization; QN – quantile normalization; QN (CN) – quantile normalization with CrossNorm; QN-Z – quantile normalization followed by z-score; TDM – Training Distribution Matching; UN – untransformed; Z – z-score)

a

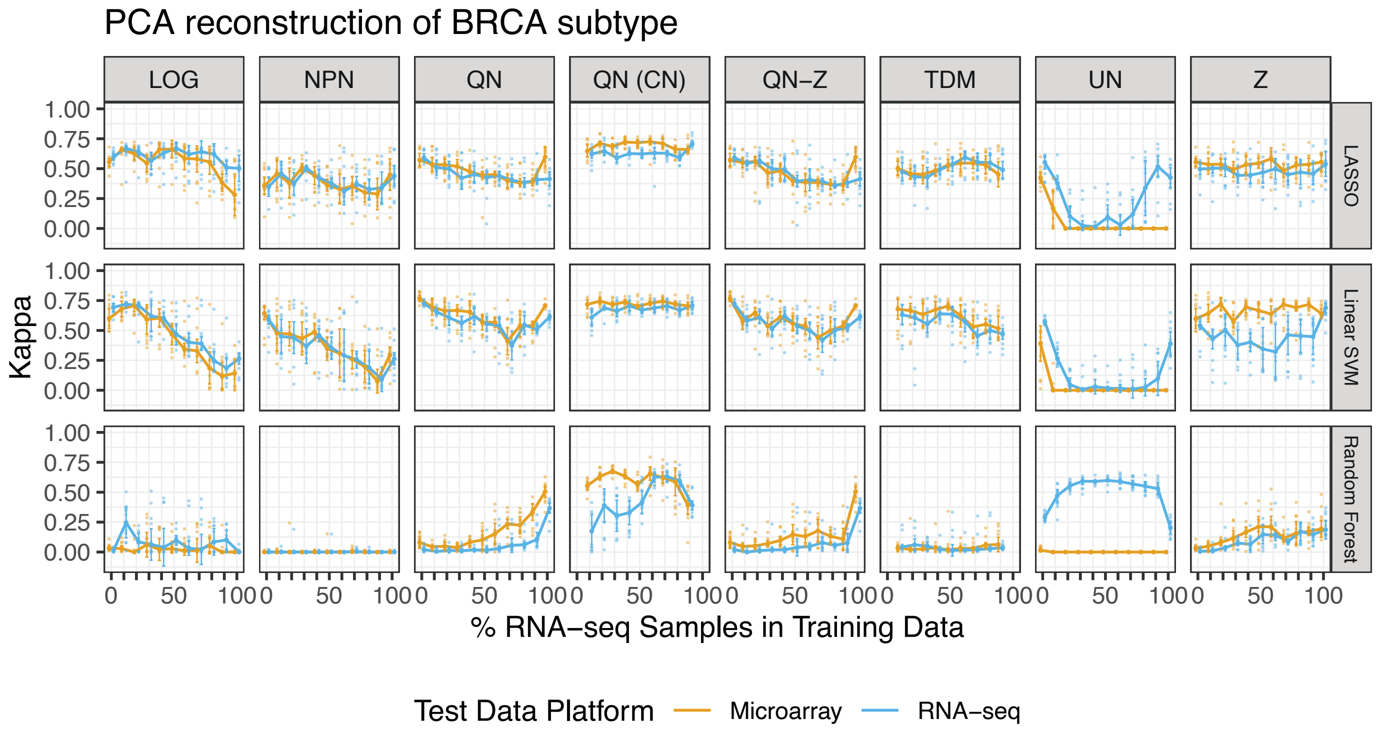

b

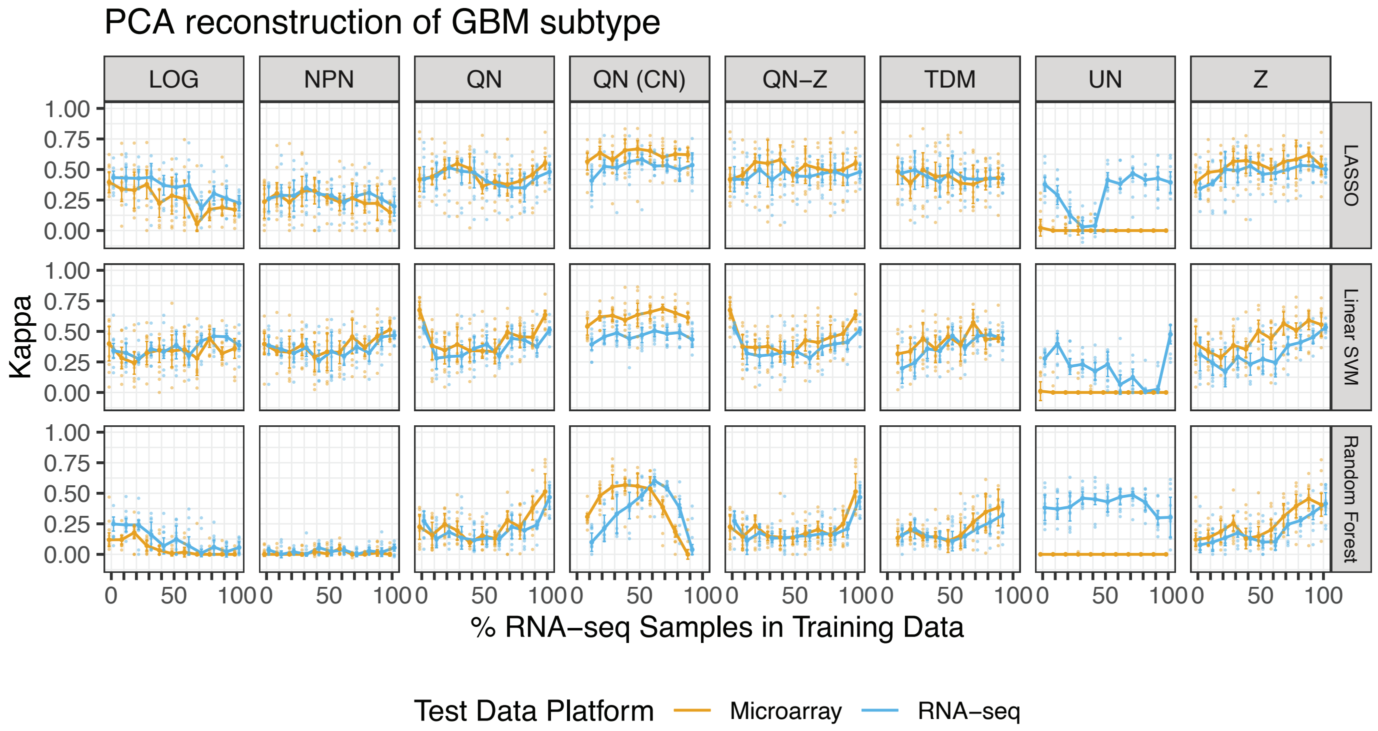

Supplementary Figure 6. Subtype classifier performance on reconstructed microarray and RNA-seq test data from (a) BRCA and (b) GBM. Median Kappa statistics from 10 repeats of steps 1-3A and 3B from Figure 1 and for seven normalization methods (and untransformed data) are displayed. Median values are shown as points, and approximate 95% confidence intervals are shown around each median defined as +/- 1.58*IQR/sqrt(n) with IQR = interquartile range and n = number of observations.[60] (LOG - log2-transformed; NPN – nonparanormal normalization; QN – quantile normalization; QN (CN) – quantile normalization with CrossNorm; QN-Z – quantile normalization followed by z-score; TDM – Training Distribution Matching; UN – untransformed; Z – z-score)

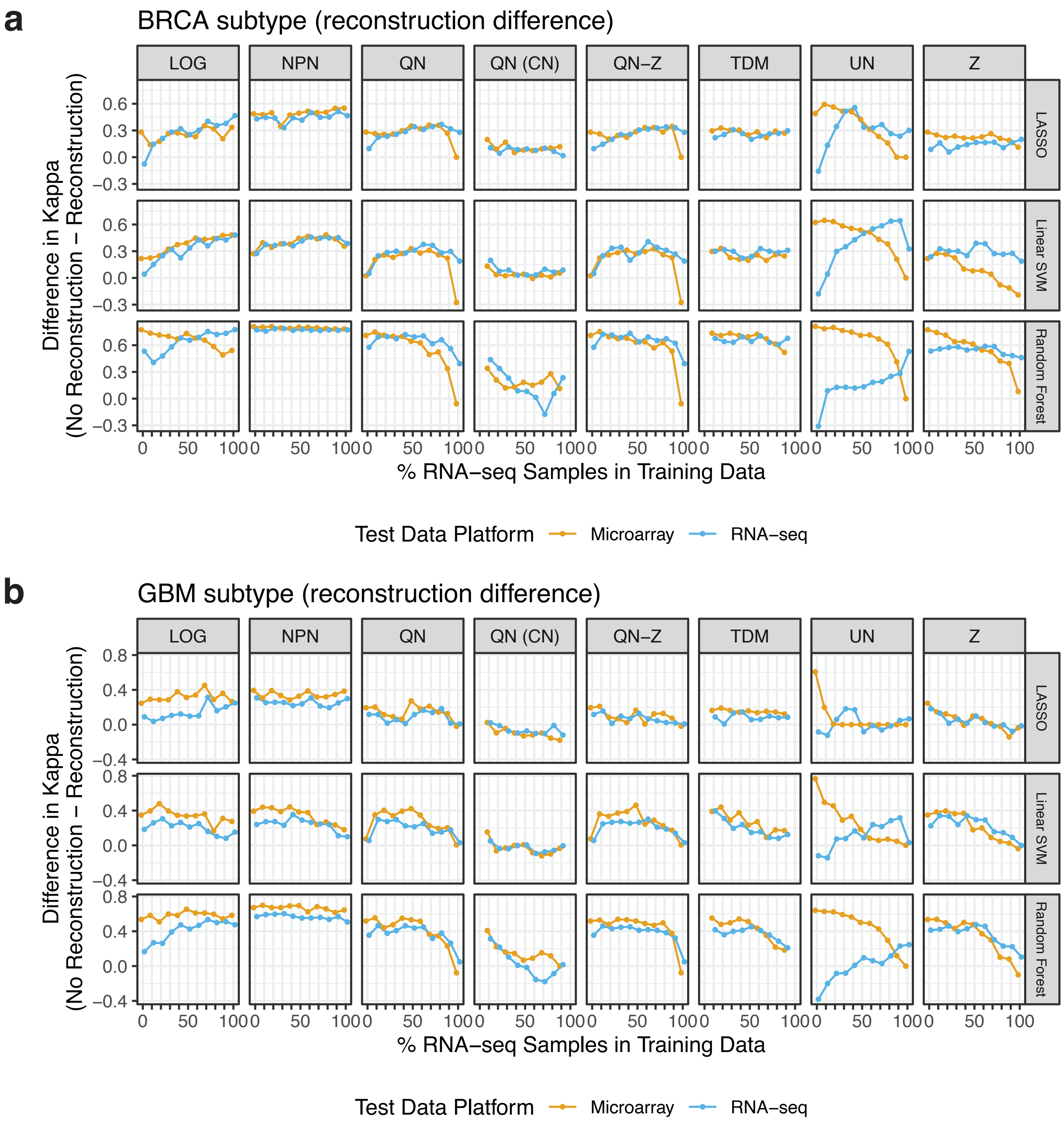

Supplementary Figure 7. Difference in subtype classifier Kappa values between non-reconstructed and reconstructed microarray and RNA-seq test data from (a) BRCA and (b) GBM. Line plots of the median difference in Kappa statistics from 10 repeats of steps 1-3A and 3B from Figure 1 and for seven normalization methods (and untransformed data) are displayed. Median values are shown as points. (LOG - log2-transformed; NPN – nonparanormal normalization; QN – quantile normalization; QN (CN) – quantile normalization with CrossNorm; QN-Z – quantile normalization followed by z-score; TDM – Training Distribution Matching; UN – untransformed; Z – z-score)

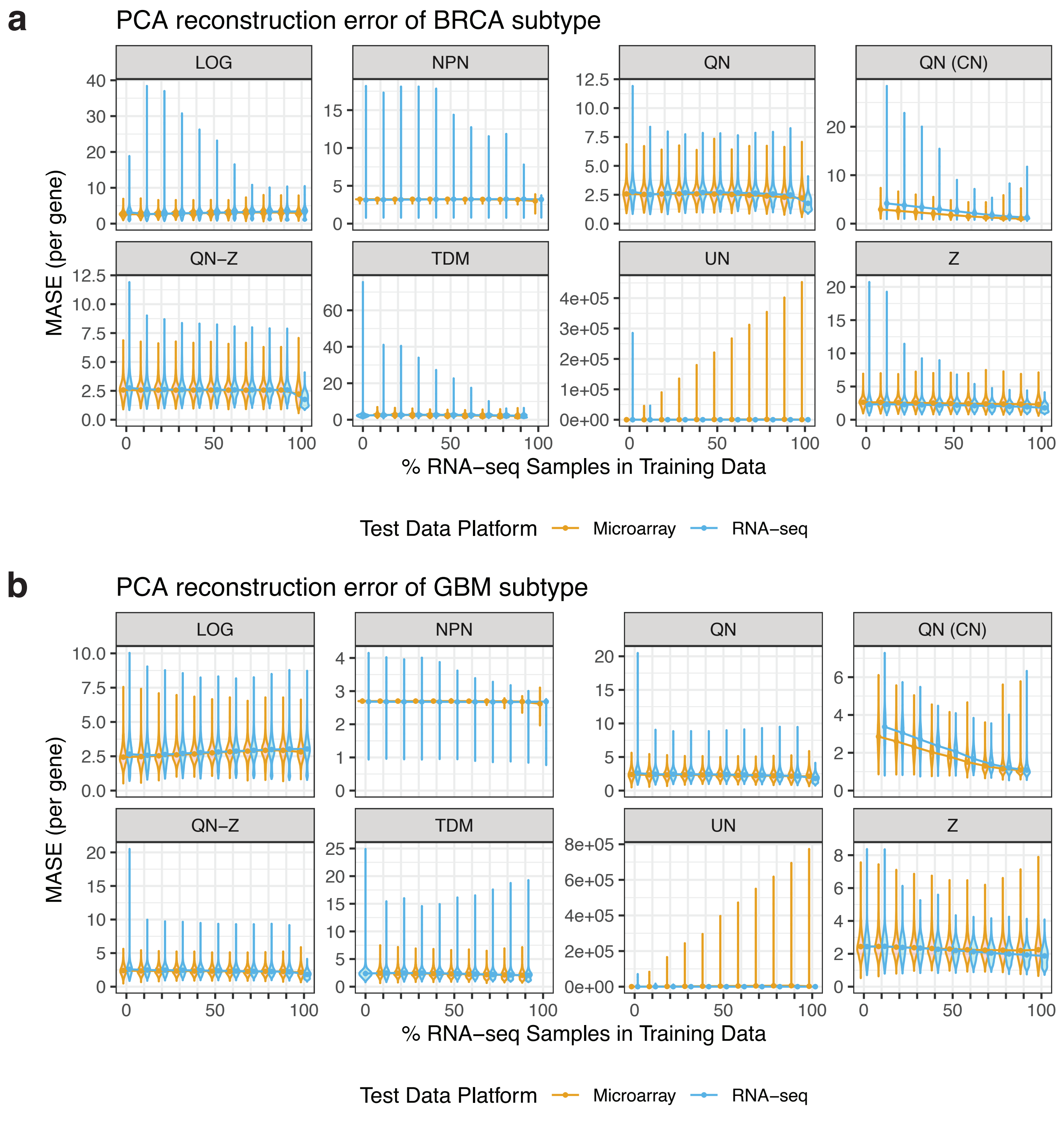

Supplementary Figure 8. Subtype classifier mean absolute squared error (MASE) on reconstructed microarray and RNA-seq test data from (a) BRCA and (b) GBM. Violin plots of per gene MASE values from 10 repeats of steps 1-3A and 3B from Figure 1 and for seven normalization methods (and untransformed data) are displayed. Median values are shown as points. (LOG - log2-transformed; NPN – nonparanormal normalization; QN – quantile normalization; QN (CN) – quantile normalization with CrossNorm; QN-Z – quantile normalization followed by z-score; TDM – Training Distribution Matching; UN – untransformed; Z – z-score)
