# Supplementary material for: Cross-platform normalization enables machine learning model training on microarray and RNA-seq data simultaneously
Source: Commun Biol. 2023 Feb 25;6:222. doi: 10.1038/s42003-023-04588-6 (PMC9968332; doi:10.1038/s42003-023-04588-6)
Supplement: Supplementary file 3 — Description of Additional Supplementary Files [file 42003_2023_4588_MOESM3_ESM.pdf]

Description of Additional Supplementary Files

File name: Supplementary Data 1

Description: Model performance metrics for each prediction task by cancer type, predictor, percentage RNA-seq, classifier used, normalization method, and test data platform

File name: Supplementary Data 2

Description: Pathway-Level Information Extractor (PLIER) results for breast cancer (BRCA), separated by normalization method (nmeth), percentage RNA-seq (pseq), and replication seed index (seed_index)

File name: Supplementary Data 3

Description: Pathway-Level Information Extractor (PLIER) results for glioblastoma (GBM), separated by normalization method (nmeth), percentage RNA-seq (pseq), and replication seed index (seed_index)
