# Supplementary material for: Cross-platform normalization enables machine learning model training on microarray and RNA-seq data simultaneously
Source: Commun Biol. 2023 Feb 25;6:222. doi: 10.1038/s42003-023-04588-6 (PMC9968332; doi:10.1038/s42003-023-04588-6)
Supplement: Supplementary file 7 — Reporting Summary [file 42003_2023_4588_MOESM7_ESM.pdf]

nature portfolio

Corresponding author(s): Casey S. Greene
Jaclyn N. Taroni

Last updated by author(s): Jan 10, 2023

Reporting Summary

Nature Portfolio wishes to improve the reproducibility of the work that we publish. This form provides structure for consistency and transparency in reporting. For further information on Nature Portfolio policies, see our Editorial Policies and the Editorial Policy Checklist.

Statistics

For all statistical analyses, confirm that the following items are present in the figure legend, table legend, main text, or Methods section.

n/a	Confirmed	
☐	☒	The exact sample size (*n*) for each experimental group/condition, given as a discrete number and unit of measurement
☐	☒	A statement on whether measurements were taken from distinct samples or whether the same sample was measured repeatedly
☐	☒	The statistical test(s) used AND whether they are one- or two-sided<br>*Only common tests should be described solely by name; describe more complex techniques in the Methods section.*
☐	☒	A description of all covariates tested
☐	☒	A description of any assumptions or corrections, such as tests of normality and adjustment for multiple comparisons
☐	☒	A full description of the statistical parameters including central tendency (e.g. means) or other basic estimates (e.g. regression coefficient) AND variation (e.g. standard deviation) or associated estimates of uncertainty (e.g. confidence intervals)
☐	☒	For null hypothesis testing, the test statistic (e.g. *F*, *t*, *r*) with confidence intervals, effect sizes, degrees of freedom and *P* value noted<br>*Give P values as exact values whenever suitable.*
☐	☒	For Bayesian analysis, information on the choice of priors and Markov chain Monte Carlo settings
☐	☒	For hierarchical and complex designs, identification of the appropriate level for tests and full reporting of outcomes
☐	☒	Estimates of effect sizes (e.g. Cohen's *d*, Pearson's *r*), indicating how they were calculated

Our web collection on statistics for biologists contains articles on many of the points above.

Software and code

Policy information about availability of computer code

Data collection	No software was used for data collection. Data used in this study are publicly available from The Cancer Genome Atlas (https://portal.gdc.cancer.gov/).
Data analysis	Source code is available on Github at https://www.github.com/greenelab/RNAseq_titration_results under a BSD-3 license. The computational environment necessary to replicate our results are available in a Docker container. Instructions for how to access the Docker container, download data, and get started running analyses are available on our Github page.

For manuscripts utilizing custom algorithms or software that are central to the research but not yet described in published literature, software must be made available to editors and reviewers. We strongly encourage code deposition in a community repository (e.g. GitHub). See the Nature Portfolio guidelines for submitting code & software for further information.

Data

Policy information about availability of data

All manuscripts must include a data availability statement. This statement should provide the following information, where applicable:
- Accession codes, unique identifiers, or web links for publicly available datasets
- A description of any restrictions on data availability
- For clinical datasets or third party data, please ensure that the statement adheres to our policy

All data that support the findings of this study are available on Figshare Plus at https://doi.org/10.25452/figshare.plus.19629864. Data and code used to create data plots are available on Figshare at https://doi.org/10.6084/m9.figshare.19686453.

Field-specific reporting

Please select the one below that is the best fit for your research. If you are not sure, read the appropriate sections before making your selection.

☒ Life sciences ☐ Behavioural & social sciences ☐ Ecological, evolutionary & environmental sciences

For a reference copy of the document with all sections, see nature.com/documents/nr-reporting-summary-flat.pdf

Life sciences study design

All studies must disclose on these points even when the disclosure is negative.

Sample size	No sample size calculation was performed. Sample sizes were determined by the public availability of matched samples processed on two gene expression platforms from The Cancer Genome Atlas breast and glioblastoma patient cohorts. Training data were defined to be two-thirds of available data from each cancer type, and test data were the remaining one-third.
Data exclusions	Patients were excluded if they did not have matched samples processed on two gene expression platforms. Genes were excluded if they were not measured on both platforms or if there was no variation in expression level.
Replication	In general, experiments were replicated 10 times using different random seeds to quantify variation in our evaluation metrics.
Randomization	Samples were randomly segregated into training and test sets such that predictor class balance was maintained.
Blinding	Blinding was not relevant to this study design as there were no treatment or control groups to consider.

Reporting for specific materials, systems and methods

We require information from authors about some types of materials, experimental systems and methods used in many studies. Here, indicate whether each material, system or method listed is relevant to your study. If you are not sure if a list item applies to your research, read the appropriate section before selecting a response.

Materials & experimental systems

n/a	Involved in the study	
☒	☐	Antibodies
☒	☐	Eukaryotic cell lines
☒	☐	Palaeontology and archaeology
☒	☐	Animals and other organisms
☒	☐	Human research participants
☒	☐	Clinical data
☒	☐	Dual use research of concern

Methods

n/a	Involved in the study	
☒	☐	ChIP-seq
☒	☐	Flow cytometry
☒	☐	MRI-based neuroimaging
